# YEATS2 O-GlcNAcylation promotes chromatin association of the ATAC complex and lung cancer tumorigenesis

**DOI:** 10.1016/j.jbc.2025.110388

**Published:** 2025-06-18

**Authors:** Jianxin Zhao, Zheng Zhao, Wen Zhou, Jianzhi Zhang, Jinfeng Chen, Jianwei Sun, Jing Li

**Affiliations:** 1 Beijing Key Laboratory of DNA Damage Response and College of Life Sciences, Capital Normal University, Beijing, China; 2 State Key Laboratory for Conservation and Utilization of Bio-Resources in Yunnan, Yunnan Key Laboratory of Cell Metabolism and Diseases, Center for Life Sciences, School of Life Sciences, Yunnan University, Kunming, China; 3 College of Chemistry and Molecular Engineering, Peking University, Beijing, China; 4 Key Laboratory of Carcinogenesis and Translational Research (Ministry of Education), Department of Thoracic Surgery II, Peking University Cancer Hospital & Institute, Beijing, China

**Keywords:** O-GlcNAc, YEATS2, ATAC, H3K9ac, ZZZ3

## Abstract

The intracellular *O*-linked β-*N*-acetylglucosamine (O-GlcNAc) modification is known to be enriched in the nucleus and on chromatin, but many of its chromatin targets remain to be identified. Herein, we demonstrate the O-GlcNAcylation of Yaf9, ENL, AF9, Taf14, Sas5 (YEATS) domain–containing 2 (YEATS2), a subunit of the chromatin Ada-two-A-containing (ATAC) complex and a reader of H3K27 acetylation levels. We show that YEATS2 interacts with the O-GlcNAc transferase and further pinpoint its major O-GlcNAcylation site to be Thr604 using electron transfer dissociation mass spectrometry. O-GlcNAcylation promotes the chromatin association of YEATS2, and the affinity between YEATS2 and other ATAC components on chromatin, such as ZZZ3, GCN5, and PCAF. Downstream, YEATS2-T604A mutants attenuated the ATAC-dependent H3K9 acetylation levels and inactivated the expression of essential ribosomal genes as shown in chromatin immunoprecipitation assays. Furthermore, xenograft experiments show that YEATS2 O-GlcNAcylation promotes lung cancer tumorigenesis. Our work reveals the critical role of YEATS2 O-GlcNAcylation in stabilizing the ATAC complex on chromatin and expands the chromatin substrates of O-GlcNAc transferase.

*O*-linked β-*N*-acetylglucosamine (O-GlcNAc) glycosylation, a reversible modification, is catalyzed by the sole O-GlcNAc transferase (OGT) and removed by the sole eraser O-GlcNAcase (1, 2). It occurs on Ser/Thr residues and is the most abundant monosaccharide modification occurring in the cytoplasm, nucleus, and mitochondria in animal cells. O-GlcNAcylation has a significant impact on various biological processes and monitors transcription, neural development, cell cycle, and stress response (1, 2).

The histone code is essential for the proper expression, maintenance, and replication of eukaryotic genomes. Accumulating evidence suggests that O-GlcNAc is part of the histone code. For instance, histones H2A, H2B, and H4 are O-GlcNAcylated, and histone O-GlcNAcylation levels oscillate during mitosis and change with heat shock (3). Histone H2B Ser112 O-GlcNAcylation facilitates its monoubiquitination (4). Histone H4 Ser47 O-GlcNAcylation is demonstrated to be important for replication origin activation (5). In addition, the eraser for histone H2AK119ub, ubiquitin-specific peptidase 16, is O-GlcNAcylated at Thr203 and Ser214, which stimulates its enzymatic activity for H2A deubiquitination (6). Moreover, the writer for histone H3K27me3, enhancer of zeste homolog 2 (EZH2), is O-GlcNAcylated and stabilized (7, 8).

In this work, we report the O-GlcNAcylation of a histone “reader”, Yaf9, ENL, AF9, Taf14, Sas5 (YEATS) domain–containing 2 (YEATS2), which is a scaffolding subunit of the ADA-two-A-containing (ATAC) complex. The ATAC complex is a transcriptional coactivator and acetylates histones at distinct residues, for example, H3K9 acetylation (H3K9ac) levels (9). It forms and functions in the nucleus, as its subunits cannot be detected in the cytoplasm (10). It contains 10 subunits. Four subunits form the histone acetyltransferase module that contains the histone acetyltransferase enzymes GCN5 or PCAF and the structural subunits SGF29, TADA3, and TADA2A (11), in which GCN5 and PCAF are mutually exclusive of each other. The six additional subunits of ATAC include YEATS2 and ZZZ3, for chromatin binding; NC2β for transcriptional regulation; WDR5 for nucleosome remodeling; and CRP2BP (11). Besides histones, ATAC also acetylates nonhistone substrates, such as cyclin A/Cdk2, to regulate cell cycle progression (12).

The YEATS domain of YEATS2 binds to acetylated histone H3K27 and recruits the ATAC complex to chromatin to write H3K9ac on promoters, which in turn maintains an open and acetylated chromatin environment to promote the expression of ribosomal protein genes and lung cancer tumorigenesis (13). Moreover, YEATS2 binds histone H3K27 crotonylation through an end-open aromatic sandwich pocket (14) and histone lysine benzoylation through its “tip-sensor” pocket (15). Recently, the peptide-based inhibitors of YEATS domains were developed by targeting a unique π–π–π stacking with expanded π systems (16). The most selective inhibitor showed a much higher binding affinity toward the AF9 YEATS domain over the ENL YEATS domain and other human YEATS domains (17), suggesting that targeting the YEATS domain could be of therapeutic value.

Herein, we show that YEATS2 is O-GlcNAcylated. Electron transfer dissociation (ETD) mass spectrometry (MS) and mutagenesis studies demonstrate that YEATS2 is mainly O-GlcNAcylated at Thr604 and the T604A mutation significantly downregulated O-GlcNAcylation levels. *Via* chromatin fractionation analysis, we found that YEATS2 O-GlcNAcylation promotes its chromatin binding and subsequent chromatin association of the ATAC complex. Chromatin immunoprecipitation (ChIP) assays further showed that ribosomal gene expression was regulated by YEATS2 O-GlcNAcylation. Finally, xenograft experiments show that YEATS2 O-GlcNAcylation promotes tumorigenesis in lung cancer. Taken together, our work suggests that YEATS2 O-GlcNAcylation promotes its chromatin association, subsequent ATAC-dependent ribosomal gene expression, and tumorigenesis of lung cancer.

## Results

### YEATS2 interacts with OGT

**Figure 1:**
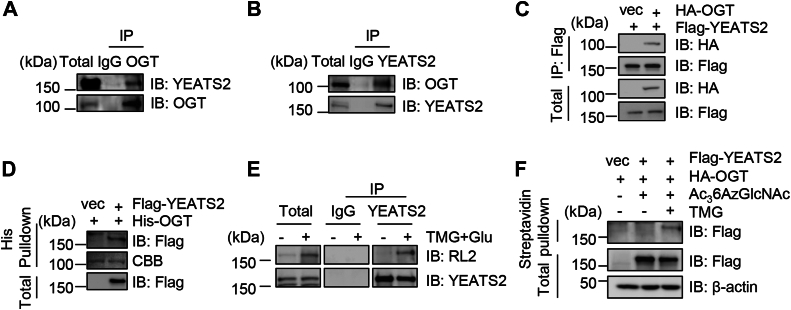
**YEATS2 interacts with OGT.***A,* 293T cells were immunoprecipitated (IPed) with anti-OGT antibodies and immunoblotted (IBed) with the indicated antibodies. *B,* 293T cells were IPed with anti-YEATS2 antibodies and IBed with the indicated antibodies. *C,* 293T cells were transfected with FLAG-YEATS2 and HA-OGT plasmids. The cell lysates were subject to immunoprecipitation and immunoblotting with the antibodies indicated. *D,* cells were transfected with FLAG-YEATS2 plasmids or vectors. Then recombinant His-OGT proteins were incubated with the cellular lysates. *E,* cells were enriched for O-GlcNAc proteins by treating with Thiamet-G (TMG) plus glucose (TMG + G) as previously described (6). The cell lysates were IPed with anti-YEATS2 antibodies and IBed with anti-O-GlcNAc RL2 antibodies. *F,* cells were transfected with FLAG-YEATS2 and HA-OGT, then treated with 200 μM Ac_3_6AzGlcNAc and 5 μM TMG. Click chemistry was then carried out as described in Experimental procedures section. CBB, Coomassie Brilliant Blue; OGT, O-GlcNAc transferase; YEATS2, Yaf9, ENL, AF9, Taf14, Sas5 domain–containing 2.

As YEATS2 has reproducibly been identified in O-GlcNAc profiling screens (oglcnac.org), we first assessed the binding affinity between YEATS2 and OGT. Cell extracts were immunoprecipitated (IPed) with anti-OGT antibodies, and the immunoprecipitates (IPs) were immunoblotted with anti-YEATS2. YEATS2 was found to co-IP with OGT (Fig. 1*A*). OGT proteins were also present in the anti-YEATS2 IPs (Fig. 1*B*). To investigate whether YEATS2 interacts with OGT exogenously, we cotransfected 293T cells with HA-OGT and FLAG-YEATS2 plasmids and performed co-IP assays with the anti-FLAG antibody. YEATS2 was also found to co-IP with OGT (Fig. 1*C*). Then pulldown assays were utilized to evaluate the physical association. 293T cells were transfected with FLAG-YEATS2 plasmids, and the cell lysates were incubated with recombinant His-OGT proteins. His-OGT pulled down overproduced YEATS2 proteins (Fig. 1*D*). These results suggest that OGT interacts with YEATS2.

We then investigated whether YEATS2 is O-GlcNAcylated. We treated cells with the OGA inhibitor, Thiamet-G (TMG), together with glucose (abbreviated at TMG + Glu), to increase the levels of protein O-GlcNAcylation (18). Under this condition, the IPed YEATS2 showed an apparent O-GlcNAc band (Fig. 1*E*). Click chemistry was also utilized to examine YEATS2 O-GlcNAcylation. We detected a crisp band upon O-GlcNAc enrichment (Fig. 1*F*). These results suggest that YEATS2 is O-GlcNAcylated.

### YEATS2 is O-GlcNAcylated at Thr-604

**Figure 2:**
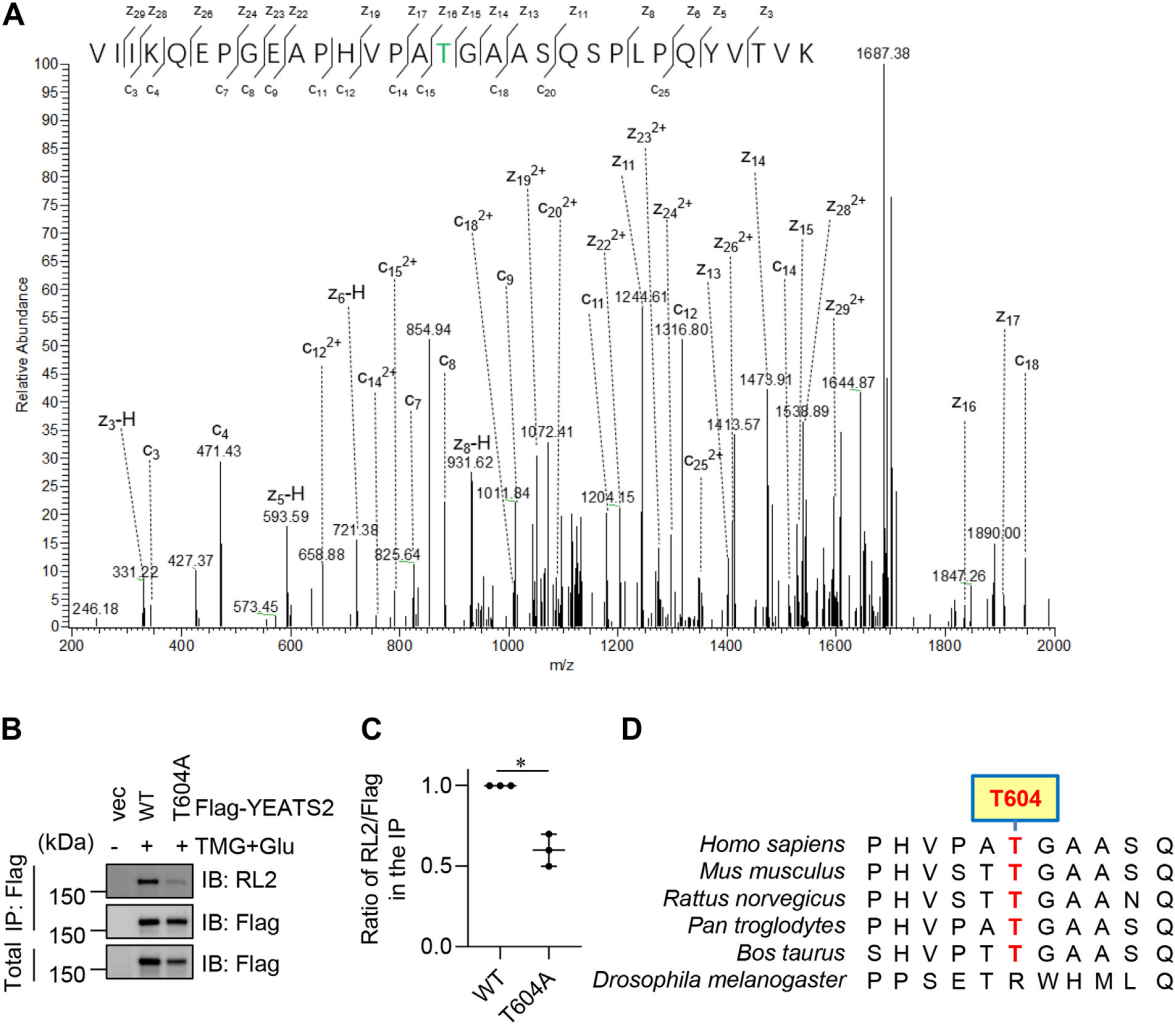
**YEATS2 is O-GlcNAcylated at T604.***A,* electron transfer dissociation mass spectrometry identified that Thr-604 is O-GlcNAcylated. *B,* cells were transfected with vector, FLAG-YEATS2-WT and -T604A plasmids, then treated with the OGA inhibitor Thiamet-G (TMG) and glucose to enrich for O-GlcNAcylation. Then the cell lysates were immunoprecipitated with anti-FLAG antibodies and immunoblotted with anti-O-GlcNAc RL2 antibodies. *C,* quantitation of (*B*). *D,* the potential O-GlcNAc site Thr-604 is conserved. Quantitation in *C* was done with a Student's *t* test. ∗ indicates *p* < 0.05. OGT, O-GlcNAc transferase; YEATS2, Yaf9, ENL, AF9, Taf14, Sas5 domain–containing 2.

To find out the sites of O-GlcNAcylation, we transfected 293T cells with FLAG-YEATS2 plasmids, and cellular lysates were IPed with anti-FLAG antibodies, and the IPs were subject to ETD MS analysis (Fig. 2*A*). MS identified a peptide modified by O-GlcNAc and suggested that Thr604 was modified (Fig. 2*A*). Then we transfected cells with FLAG-YEATS2 or FLAG-YEATS2-T604A plasmids and treated cells with TMG and glucose. We observed that mutations at Thr604 (T604A) greatly diminished RL2 signals, suggesting that this site was modified (Fig. 2, *B* and *C*). Sequence alignment shows that YEATS2 Thr604 is conserved (Fig. 2*D*). Taken together, our results suggest that YEATS2 is O-GlcNAcylated, and the major modification site is Thr604. Our results do not exclude the possibility that YEATS2 may contain other O-GlcNAc sites.

### YEATS2 O-GlcNAcylation stimulates its chromatin binding

**Figure 3:**
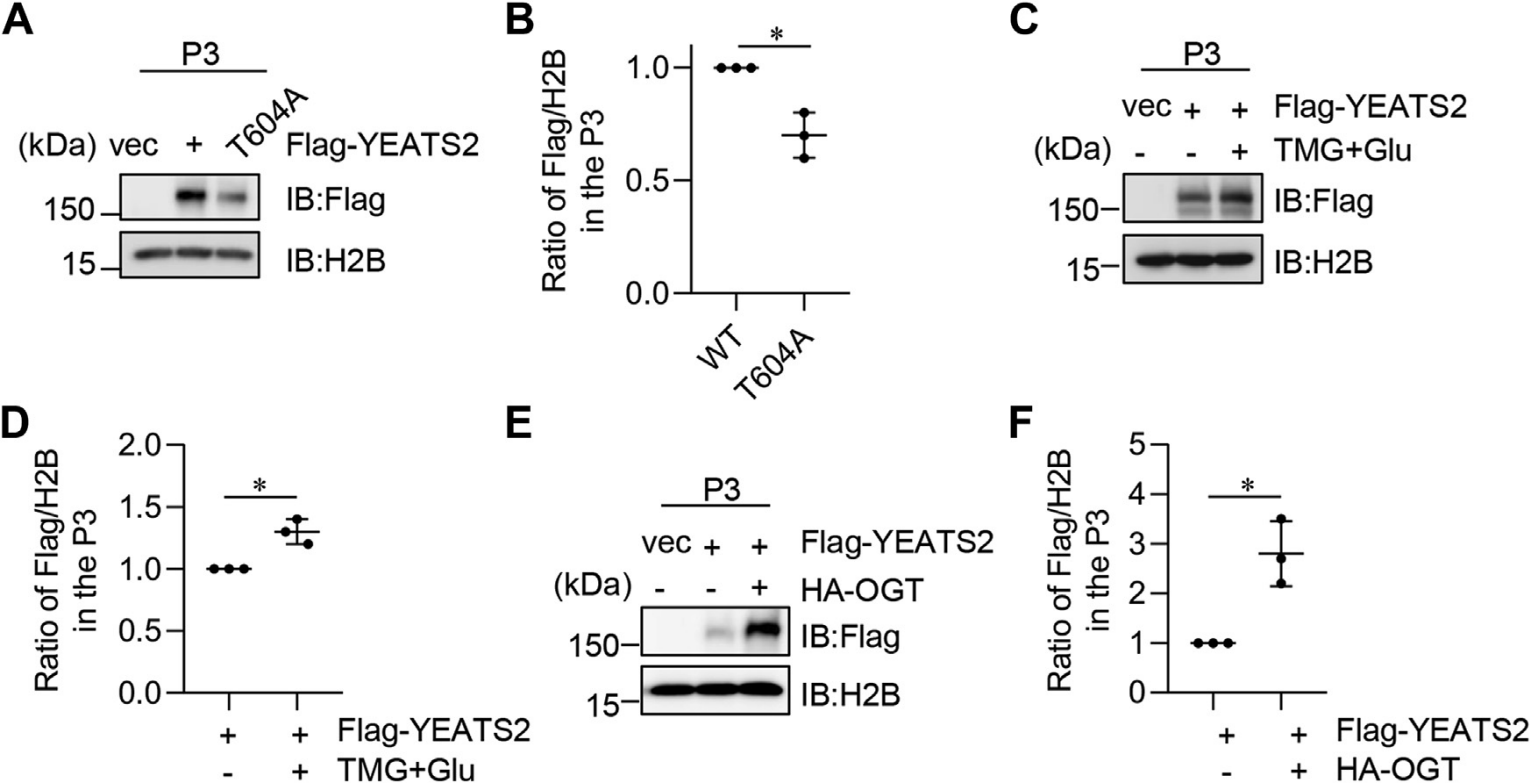
**YEATS2 O-GlcNAcylation promotes its chromatin binding.***A,* cells were transfected with vectors, FLAG-YEATS2-WT, or -T604A plasmids, and the cell lysates were fractionated into chromatin portions (P3). Immunoblotting was carried out to identify chromatin-binding proteins. *B,* quantitation of the results in (*A*). *C,* cells were treated or untreated with TMG and glucose, and then the cell lysates were fractionated into chromatin portions and immunoblotted with indicated antibodies. *D,* quantitation of the results in (*C*). *E,* cells were transfected with FLAG-YEATS2 and HA-OGT plasmids. Chromatin fractionation was carried out. *F,* quantitation of (*E*). A Student's *t* test was used in (*B*), (*D*), and (*F*). ∗ indicates *p* < 0.05. TMG, Thiamet-G; YEATS2, Yaf9, ENL, AF9, Taf14, Sas5 domain–containing 2.

The YEATS domain of YEATS2 binds to acetylated histone H3K27, and all the YEATS domain proteins are associated with chromatin-associated complexes (19). Therefore, we investigated whether O-GlcNAcylation regulates the chromatin affinity of YEATS2. We transfected FLAG-YEATS2-WT and -T604A plasmids into 293T cells. In the chromatin fractionation analysis, we found that the T604A mutation, which abolishes its O-GlcNAcylation, caused a significant decrease of YEATS2 in the chromatin fraction (Fig. 3, *A* and *B*). We then treated cells with TMG and Glu and examined its effect on the chromatin affinity of WT YEATS2. As shown in Figure 3, *C* and *D*, the level of YEATS2 on chromatin was significantly increased in the TMG plus Glu-treated lane compared with the control, suggesting that YEATS2 O-GlcNAcylation promotes its chromatin binding. We also overproduced OGT to examine the association between YEATS2 and chromatin (Fig. 3, *E* and *F*). Consistently, OGT overproduction elevated chromatin association of YEATS2. These findings indicate that O-GlcNAcylation enhances chromatin-bound YEATS2.

### YEATS2 O-GlcNAcylation promotes ATAC complex chromatin binding

**Figure 4:**
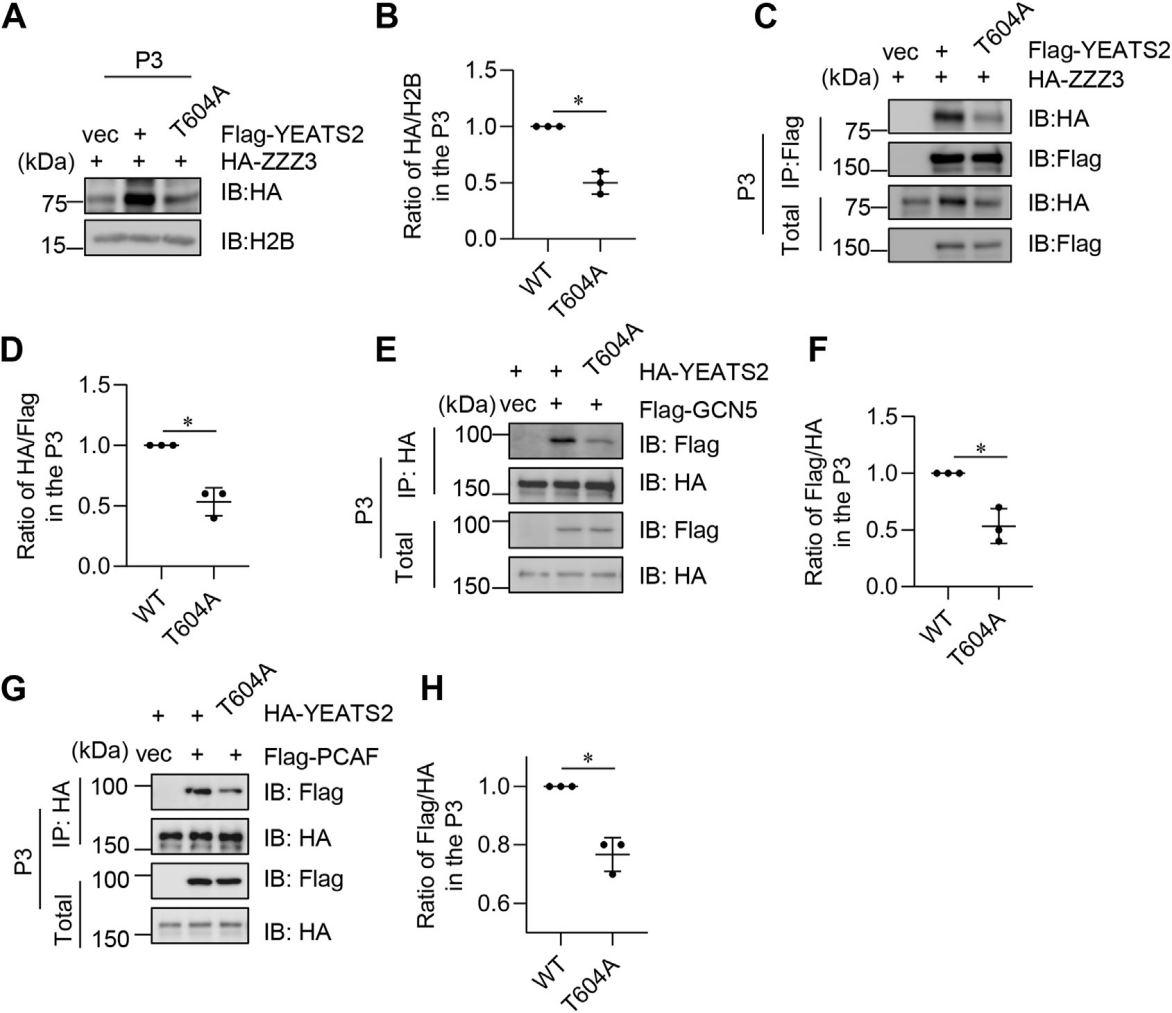
**YEATS2 O-GlcNAcylation promotes ATAC complex chromatin binding.***A,* cells were transfected with vectors, FLAG-YEATS2-WT, or -T604A and HA-ZZZ3 plasmids. Then the cell lysates were fractionated into chromatin portions and immunoblotted (IBed) with anti-HA antibodies. *B,* quantitation of the results in (*A*). *C,* cells were transfected with vectors, FLAG-YEATS2-WT, or -T604A and HA-ZZZ3 plasmids. Then the cell lysates were fractionated into chromatin portions and immunoprecipitated (IPed) with anti-FLAG antibodies and IBed with indicated antibodies. *D,* quantitation of the results in (*C*). *E,* cells were transfected with vectors, HA-YEATS2-WT, or -T604A and FLAG-GCN5 plasmids, and the cell lysates were fractionated into chromatin portions. Then the cell lysates were IPed with anti-HA antibodies and IBed with indicated antibodies. *F,* quantitation of the results in (*E*). *G,* cells were transfected with vectors, HA-YEATS2-WT, or -T604A and FLAG-PCAF, and the cell lysates were fractionated into chromatin portions. Then the cell lysates were IPed with anti-HA antibodies and IBed with indicated antibodies. *H,* quantitation of the results in (*G*). A Student's *t* test was used in (*B*), (*D*), (*F*), and (*H*). ∗ indicates *p* < 0.05. ATAC, Ada-two-A-containing; YEATS2, Yaf9, ENL, AF9, Taf14, Sas5 domain–containing 2.

As YEATS2 is a scaffolding subunit of the ATAC complex (11), we tested the role of YEATS2 O-GlcNAcylation in the chromatin affinity of the ATAC complex. We first examined the chromatin recruitment of ZZZ3 and found that FLAG-YEATS2-T604A would significantly decrease the chromatin fraction of ZZZ3 (Fig. 4, *A* and *B*). To investigate the interaction between YEATS2 and ZZZ3 on chromatin, we cotransfected 293T cells with HA-ZZZ3 and FLAG-YEATS2-WT or -T604A plasmids and performed co-IP assays on the chromatin fractionation. The T604A mutant significantly diminished the interaction between YEATS2 and ZZZ3 on chromatin (Fig. 4, *C* and *D*). These results suggest that YEATS2 O-GlcNAcylation upregulates the association between ZZZ3 and YEATS2 on chromatin.

Then we assessed the affinity between YEATS2 and other ATAC components, such as GCN5 and PCAF, two histone acetyltransferases. We found that the T604A mutant diminished the interaction between YEATS2 and GCN5 on chromatin (Fig. 4, *E* and *F*) as well as PCAF (Fig. 4, *G* and *H*). Together, these data suggest that YEATS2 O-GlcNAcylation promotes the chromatin association of the ATAC complex.

### YEATS2 O-GlcNAcylation upregulates the expression of ATAC-dependent ribosomal genes

**Figure 5:**
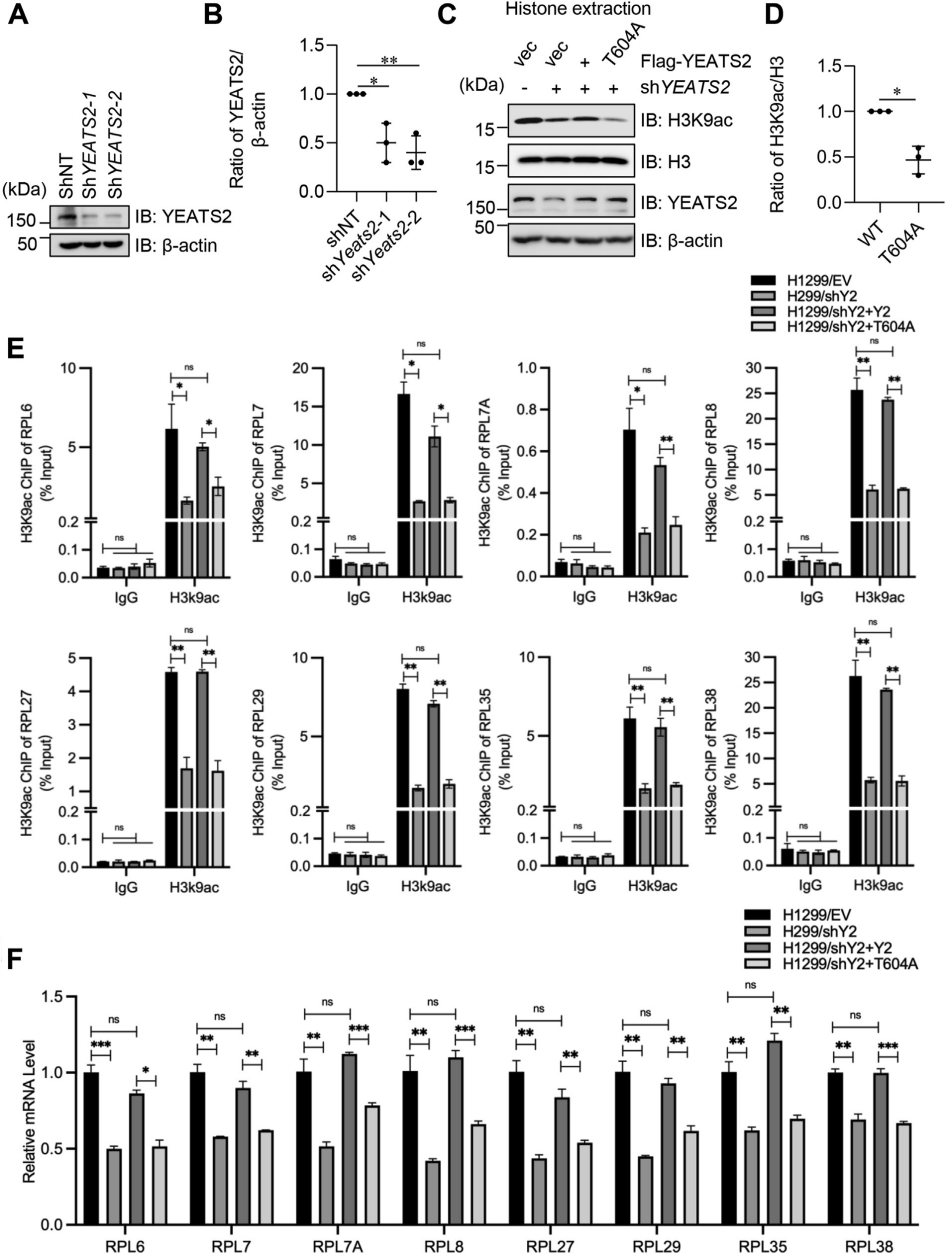
**YEATS2 O-GlcNAcylation promotes ATAC-dependent ribosomal protein gene expression.***A,* stable *YEATS2*-knockdown H1299 cell lines were generated using two independent siRNA oligos targeting YEATS2 and examined for YEATS2 expression. *B,* quantitation of (*A*). *C,* the cell lines in (*A*) were rescued with FLAG-YEATS2-WT or -T604A plasmids. Then the cellular histone was extracted and immunoblotted with indicated antibodies. *D,* quantitation of (*C*). *E,* qPCR analysis of H3K9ac chromatin immunoprecipitation (ChIP) in the promoters of the indicated ribosomal protein genes in sh*YEATS2* H1299 cells ectopically expressing YEATS2-WT or the T604A mutant. *F,* qRT–PCR analysis of the expression of ribosomal protein genes in cells as in (*E*). Quantitation in (*B*) was done with a one-way ANOVA in (*D*–*F*) with a Student's *t* test. ∗ indicates *p* < 0.05. ∗∗ indicates *p* < 0.01. ∗∗∗ indicates *p* < 0.001. ATAC, Ada-two-A-containing; ns, not significant; H3K9ac, H3K9 acetylation; qPCR, quantitative PCR; YEATS2, Yaf9, ENL, AF9, Taf14, Sas5 domain–containing 2.

It is known that ATAC regulates the expression of genes involved in ribosome biogenesis through H3K9ac in lung cancer (13). Therefore, we investigated whether YEATS2 O-GlcNAcylation regulates ATAC-dependent ribosomal gene expression. First, we generated H1299 cells stably knocking down *YEATS2* using two independent shRNAs targeting *YEATS2* (Fig. 5, *A* and *B*). Then, one knocked-down cell line was rescued with YEATS2-WT or -T604A plasmids. sh*YEATS2* significantly reduced the level of H3K9ac as reported (13). In the rescue cells, the H3K9ac level is comparable to the control in the YEATS2-WT rescued cells but not in the -T604A rescued cells (Fig. 5, *C* and *D*), suggesting that T604 O-GlcNAcylation is fundamental to ATAC-mediated H3K9ac.

Then we carried out quantitative PCR (qPCR) analysis of H3K9ac ChIP in the promoters of the indicated ribosomal genes, and we found that T604A reduced H3K9ac in all genes examined: *RPL6*, *RPL7*, *RPL7A*, *RPL8*, *RPL27*, *RPL29*, *RPL35*, and *RPL38* (Fig. 5*E*). We also assessed the expression of H3K9ac target genes that encode ribosomal proteins, and we found that the ribosomal gene expression was significantly attenuated in T604A cells (Fig. 5*F*). Taken together, YEATS2 O-GlcNAcylation upregulates ATAC-dependent ribosomal gene expression.

### YEATS2 O-GlcNAcylation promotes lung cancer

**Figure 6:**
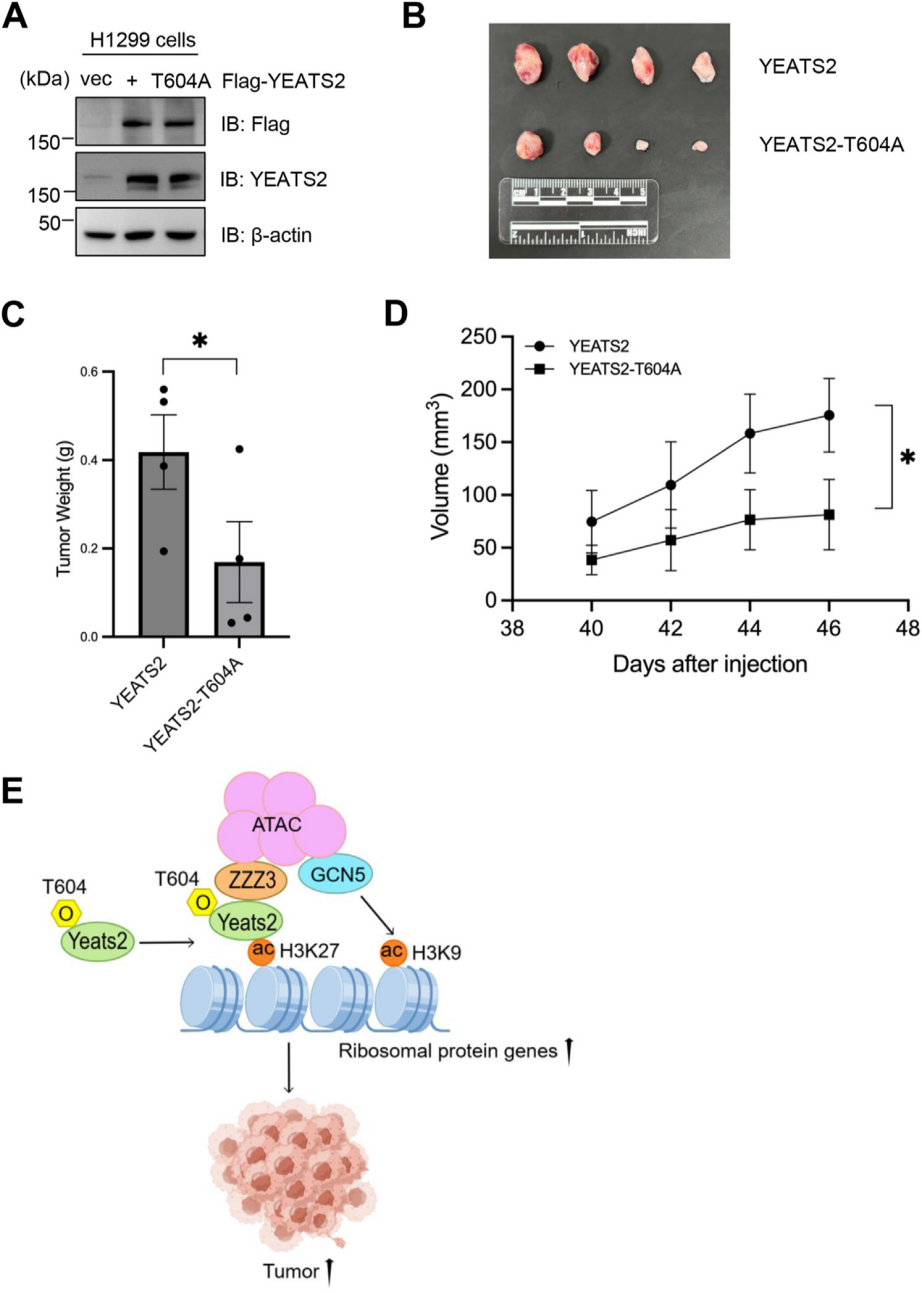
**YEATS2 O-GlcNAcylation promotes tumor cell growth.***A,* H1299 cell lines stably overexpressing YEATS2-WT or T604A plasmids were generated and examined for YEATS2 expression. *B*–*D,* the cells in (*A*) were subject to xenograft assays. Xenograft tumors formed at 46 days after subcutaneous transplantation were examined. *B,* the tumor images. *C,* the tumor weight. *D,* the tumor volume. ∗ indicates *p* < 0.05. *E,* a model illustrating the role of YEATS2 O-GlcNAcylation in tumorigenesis. O-GlcNAcylation of YEATS2 at T604 promotes its binding to chromatin, thus enhancing the chromatin recruitment of the ATAC complex and resultant H3K9ac of ribosome genes, further promoting lung cancer. By FigDraw (https://www.figdraw.com/static/index.html). ATAC, Ada-two-A-containing; H3K9ac, H3K9 acetylation; YEATS2, Yaf9, ENL, AF9, Taf14, Sas5 domain–containing 2.

It is known that YEATS2 links histone acetylation with lung cancer tumorigenesis (13). We then examined the *in vivo* effect of YEATS2 O-GlcNAcylation. We generated stable H1299 cells that overproduce YEATS2-WT or -T604A and then carried out mouse xenograft experiments (Fig. 6, *A* and *B*), and the tumor size and weight were monitored (Fig. 6, *C* and *D*). As expected, the YEATS2-WT cells produced much larger tumors compared with the T604A mutants, suggesting that YEATS2 O-GlcNAcylation enhances lung cancer, probably *via* promoting H3K9ac and its targeting genes.

## Discussion

In this work, we present evidence that YEATS2 is O-GlcNAcylated. O-GlcNAc promotes the chromatin association of not only YEATS2 but also other ATAC subunits, such as ZZZ3, GCN5, and PCAF, thereby elevating downstream histone H3K9ac (Fig. 6*E*).

Of all the ATAC complex subunits, only YEATS2 has been identified in O-GlcNAc profiling works, and multiples sites of YEATS2 were modified (oglcnac.org). Using ETD MS, we found that YEATS2 is indeed O-GlcNAcylated, with Thr604 to be one major O-GlcNAc site. Our work does not exclude the possibility that YEATS2 is O-GlcNAcylated at other residues, which may exert the same or different functions as Thr604 on YEATS2.

O-GlcNAc is an integral part of the histone code (2, 20), as numerous histone readers, erasers, and the histone itself have been identified to be O-GlcNAc substrates. Through regulating the histones, O-GlcNAc modulates gene expression, tumorigenesis, and circadian rhythm (21). In zebrafish models, circadian cycle correlates with histone H2B O-GlcNAcylation together with histone H3 trimethylation in the zebrafish brain (21). Considering that O-GlcNAc is most abundant in the human brain, it is tempting that it may have comparable effects in humans.

As newly identified histone readers for Kac and Kcr, YEATS domain proteins have been implicated in various human diseases, in particular, ENL and AF9 in acute myeloid leukemia (17). YEATS domains are therefore of great therapeutic potential (22). However, in our MS analysis of YEATS2 O-GlcNAcylation, none of the modified sites reside in the YEATS domain (amino acids 231–330). This is consistent with other O-GlcNAc studies, showing that O-GlcNAc occurs often in intrinsically disorder regions rather than in folded protein domains, which may impede its translational studies. Other YEATS domain proteins have also shown up in O-GlcNAc profiling works, such as ENL and AF9. It would be worthwhile to validate their modification sites and explore their functions.

Besides lung cancer, YEATS2 is also found to regulate other kinds of cancer, such as pancreatic cancer (23, 24), hepatocellular carcinoma (25, 26), and head and neck squamous cell carcinoma (27). Whether YEATS2 O-GlcNAcylation plays a role in these cancer types warrants further investigation. In sum, we report the O-GlcNAcylation of a histone reader, YEATS2, and its physiological role in lung cancer. O-GlcNAc may finetune other epigenetic regulators that await discovery.

## Experimental procedures

### Cell culture, antibodies, and plasmids

Cells were purchased from American Type Culture Collection. The cell lines were validated using short tandem repeat profiling and free from mycoplasma contamination for all experiments. Antibodies: anti-OGT (Abcam; catalog no.: AB96718); anti-YEATS2 (Proteintech; catalog no.: 24717-1-AP); anti-HA (Bethyl Laboratories; catalog no.: A190-108A); anti-FLAG (Sigma; catalog no.: F1084); anti-RL2 (Abcam; catalog no.: AB2739); anti-β-actin (Sigma; catalog no.: A5441); anti-H2B (Cell Signaling; catalog no.: 53H3); anti-H3K9ac (Abcam; catalog no.: AB32129); and anti-H3 (Abcam; catalog no.: ab18521). *YEATS2-T604A* plasmids were generated using specific primers (sequences available upon request) following the manufacturer's instructions (ClonExpress Ultra One Step Cloning Kit; Vazyme C115). sh*YEATS2-1:* TCAAAGAACTTGGTCATAAAT, sh*YEATS2-2:* GCACAGAAACTGACTTCTTTA.

### IP and immunoblotting assays

IP and immunoblotting experiments were performed as described before (18, 28). The following primary antibodies were used for immunoblot: anti-OGT (1:1000), anti-YEATS2 (1:1000), anti-HA (1:1000), and anti-FLAG M2 (1:1000), anti-RL2 (1:1000), anti-β-actin (1:3000), anti-H2B (1:1000), anti-H3K9ac (1:500), and anti-H3 (1:1000). Peroxidase-conjugated secondary antibodies were from Jackson ImmunoResearch. Blotted proteins were visualized using the ECL detection system (Amersham). Signals were detected by a LAS-4000 and quantitatively analyzed by densitometry using the Multi Gauge software (Fujifilm). All Western blots were repeated for at least three times.

### Cell culture treatment

Chemical utilization: TMG (OGA inhibitor) was used at 5 μM for 24 h; glucose (Glu) was used at 30 mM for 3 h.

### Click chemistry

Cells were transfected with FLAG-YEATS2 and HA-OGT plasmids and then treated with 200 μM Ac_3_6AzGlcNAc and 5 μM TMG (Sigma–Aldrich) for 24 h. Collected cells were lysed with 150 mM lysis buffer (150 mM NaCl, 1 M Tris–HCl [pH 7.5], 0.5 M EDTA, and 10% NP-40) containing a protease inhibitor cocktail (Roche) for 1 h at 4 °C. Next, cell lysates were cleared using centrifugation (4 °C; 12,000 rpm; 10 min). The supernatant was incubated with 50 μM DBCO–PEG4–Biotin from Duyouyou Biotechnology, 8 mM urea, 10 m M Hepes (pH 7.9), and Halt protease and phosphatase inhibitor cocktail (100×) from Thermo Fisher Scientific, then the pull-down complex was isolated by streptavidin-coupled beads, and subjected to Western blotting analysis.

### Chromatin fractionation

Cells were lysed in 200 μl of buffer A (10 mM Hepes, pH 7.9, 0.34 M sucrose, 1 mM DTT, 10% glycerol, 1.5 mM MgCl_2_, and freshly added protease inhibitors) and added Triton X-100 on ice for 5 min, and then centrifuged at 1500*g* for 4 min. The sediment was washed once with buffer A and centrifuged at 1500*g* for 4 min. Then the sediment was lysed in 200 μl of buffer B (3 mM EDTA, 0.2 mM EGTA, 1 mM DTT, and freshly added protease inhibitors) at 4 °C for 10 min and then centrifuged at 2000*g* for 4 min. The pellet was washed once with buffer B and centrifuged at 13,000*g* for 1 min. The pellet was boiled in SDS sample buffer.

### Histone extraction

Cells were lysed for 10 min in 500 μl of TEB buffer (0.5% Triton X-100, 1× PBS) on ice for 10 min. The lysed cells were pelleted by centrifugation at 2000× rpm for 10 min. The pellet was washed once with TEB buffer, resuspended in 0.2 M HCl, and incubated at 4 °C for overnight. HCl-containing samples were neutralized with 1 M NaOH before SDS-PAGE.

### ETD MS

The gel band pieces were dehydrated in acetonitrile, incubated in 10 mM DTT in 50 mM ammonium bicarbonate at 56 °C for 40 min, incubated in 55 mM iodoacetamide in 50 mM ammonium bicarbonate at ambient temperature for 1 h in the dark, and dehydrated again. Then the gel pieces were digested in-gel with 2 ng/μl sequencing-grade trypsin in 50 mM ammonium bicarbonate overnight at 37 °C. The resulting peptides were extracted twice with 5% formic acid (FA)/50% acetonitrile and then vacuum-centrifuged to dryness. All samples were resuspended in 0.1% FA in water prior to LC–MS/MS analysis.

Peptides were separated using a loading column (100 μm × 2 cm) and a C18 separating capillary column (75 μm × 15 cm) packed in-house with Luna 3 μm C18(2) bulk packing material (Phenomenex). The mobile phases (A: water with 0.1% FA and B: 100% acetonitrile with 0.1% FA) were driven and controlled by an EASY-nLC 1000 system (Thermo Fisher Scientific). The LC gradient was held at 2% for 1 min of the analysis, followed by an increase from 2% to 7% B from 1 to 2 min, an increase from 7% to 35% B from 2 to 62 min, and an increase from 35% to 75% B from 62 to 66 min.

The MS data were acquired in data-dependent mode with a full MS scan (300–1700 *m/z*) in Fourier transform mode at a resolution of 60,000 followed by ETD MS/MS scans on the 10 most abundant ions with multiple charges in the initial MS scan. Automatic gain control targets were 1e6 ions for Orbitrap scans and 5e4 for MS/MS scans. For dynamic exclusion, the following parameters were used: isolation window, 2 *m/z*; repeat count, 1; repeat duration, 25 s; and exclusion duration, 25 s. The ETD activation time was 150 ms. Charge state dependent time and supplemental activation for ETD were enabled.

Data processing was carried out using Thermo Proteome Discoverer 2.4 using a SwissProt *Homo sapiens* database (TaxID = 9606 and subtaxonomy, 42,253 protein sequences). Carbamidomethyl (Cys) were chosen as static modification, and oxidation (Met) and HexNAc (Ser or Thr) were chosen as variable modification. Mass tolerance was 10 ppm for precursor ions and 0.6 Da for fragment ions. Maximum missed cleavages was set as 2. Peptide spectral matches were validated using the Percolator algorithm, based on *q* values at a 1% false discovery rate at both peptide and protein levels.

### ChIP and ChIP–qPCR analysis

Cells were crosslinked with 1% formaldehyde for 15 min in room temperature and stopped with 125 mM glycine. The cells were resuspended using lysis buffer, and DNA was sonicated to let its length in 100 to 500 bp. Protein A/G Plus-agarose beads were balanced in lysis buffer. Took one-fifth sample as input, the rest four-fifths were IP. The IP samples were IPed with 6 μl of the H3K9ac antibodies (ab10812; abcam) and Protein A/G Plus-agarose beads overnight at 4 °C. The IPs were washed once each with low salt, high salt, LiCl buffer, and TE buffer. Eluted DNA and 5 M NaCl was added to both input sample and IP input sample, and the DNA was reverse crosslinked in 65 °C. Finally, the DNA was extracted by phenol–chloroform.

For ChIP–qPCR, the fluorescence signals were analyzed with a CFX96 real-time PCR detection system (Bio-Rad) using the 2× Relab Green PCR Fast Mixture (Universal) Kit (R0202-02; LABLEAD). Relative expression levels of target genes were calculated with the ΔCT method using the detected gene in input sample as an endogenous reference gene for internal normalization. The sequence of primers is listed in Table S1.

### Mouse xenograft

H1299 cells were infected with lentivirus of pLenti-CMV-Blasticidin-FLAG-Empty, pLenti-CMV-Blasticidin-FLAG-YEATS2, and pLenti-CMV-Blasticidin-FLAG-YEATS2-T604A, followed by selection with blasticidin (10 μg/ml) to establish stable H1299 cell lines expressing VEC, YEATS2, and YEATS2-T604A. Six-week-old nude mice received a single subcutaneous injection of 1.0 × 10^6^ cells in right flanks. Tumor volumes were monitored from day 40 to day 46 postinoculation, calculated using the formula: volume = ([4 × π/3] × [L/2] × [W/2] × [D])/2. On day 46, tumors were excised, and their weights were recorded during necropsy. The mice were obtained from the Animal Research and Resource Center, Yunnan University, with the certification no.: SCXK(Dian) K2021-0001. All procedures involving animal subjects were approved by the Animal Care Committee of Yunnan University.

## Data availability

The MS proteomics data have been deposited to the ProteomeXchange Consortium *via* the PRIDE (29) partner repository with the dataset identifier PXD060970 and 10.6019/PXD060970.

## Supporting information

This article contains supporting information.

## Conflict of interest

The authors declare that they have no conflicts of interest with the contents of this article.
