# Supplementary material for: YEATS2 O-GlcNAcylation promotes chromatin association of the ATAC complex and lung cancer tumorigenesis
Source: J Biol Chem. 2025 Jun 18;301(7):110388. doi: 10.1016/j.jbc.2025.110388 (PMC12275198; doi:10.1016/j.jbc.2025.110388)
Supplement: Table S1 [file mmc1.docx]

**Supplementary Table 1. Primers used in this study.**

| Primers | Sequences |
| --- | --- |
| YEATS2 RT F | AACCCCAGGGTCTGAATTTATTG |
| YEATS2 RT R | CGGACGCATACACGTTCCT |
| RPL6 RT F | CATTACCCCCGGGACCAT |
| RPL6 RT R | CTTCAGGAAAACCACCCTCTTG |
| RPL7 RT F | AGTGAGCCCAAAGGTTCGAA |
| RPL7 RT R | AGCTTCACAAAGGTTCCATTGAA |
| RPL7A RT F | GGACATCCAGCCCAAAAGAG |
| RPL7A RT R | TGCCGCTGCAACCTGATATA |
| RPL8 RT F | GGCCCAGCTCAACATTGG |
| RPL8 RT R | CCAGGCAGCACACGATTGTA |
| RPS15 RT F | GAGAAGCCGGAAGTGGTGAA |
| RPS15 RT R | GTTGAAGGTCTTGCCGTTGTAGA |
| RPL26 RT F | TGGACAAAGACCGCAAAAAGA |
| RPL26 RT R | TTTGCCCTTTTCCTTTCCTACTT |
| RPL27 RT F | ACCTGGGAAGGTGGTGCTT |
| RPL27 RT R | TTCTTCACGATGACAGCTTTGC |
| RPL29 RT F | GAACCACACCACACACAACCA |
| RPL29 RT R | CCCCCTTAAGAGATTCGTATCTTTG |
| RPL35 RT F | GCGCGTCGCCAAAGTG |
| RPL35 RT R | ACACGGGCAATGGATTTCC |
| RPL38 RT F | TGCCTCGGAAAATTGAGGAA |
| RPL38 RT R | TCTTGACAGATTTGGCATCCTTT |
| RPL6-ChIP-F | GGCATTCTACCTCACCCTCTTTG |
| RPL6-ChIP-R | GCCTTCCAGACGCTTCATTT |
| RPL7-ChIP-F | CGACGGGTTCCACACACAT |
| RPL7-ChIP-R | AGGACGGAGGTTTTGGAGATC |
| RPL7A-ChIP-F | GTTCTGATTCCTGCCACTTCACT |
| RPL7A-ChIP-R | CGGTATTTTAGAGAGGAGAGGATGTG |
| RPL8-ChIP-F | CCGCGATGCTAACCCTTCT |
| RPL8-ChIP-R | CCGCCGAGGGCATTC |
| RPS15-ChIP-F | GCGATGAGGATGCCGATT |
| RPS15-ChIP-R | GCGTCCTCCTTCCTCTTGAA |
| RPL26-ChIP-F | AAGAACGGATGGCTGCTGAT |
| RPL26-ChIP-R | AGCGGGAGCGGGTAAGG |
| RPL27-ChIP-F | CCAAGGCGACAGTGAACACA |
| RPL27-ChIP-R | AGCTGACTCCTGCCAGCAA |
| RPL29-ChIP-F | GCCATCCCCCTCCTAGGA |
| RPL29-ChIP-R | ACTGGTGACCGACCGTGTGT |
| RPL35-ChIP-F | CAGGTCGTCCAGCTGTTTCA |
| RPL35-ChIP-R | GCCAAGATCAAGGCTCGAGAT |
| RPL38-ChIP-F | TGCAGCCTCGGAAAATTGAG |
| RPL38-ChIP-R | CACTTACATTTGGCATCCTTTCG |
